# P265: Structure for prevention of healthcare-associated infection in Brazilian Hospitals

**DOI:** 10.1186/2047-2994-2-S1-P265

**Published:** 2013-06-20

**Authors:** MC Padoveze, CMCB Fortaleza, C Kiffer, AL Barth, ICRS Carneiro, JLN Rodrigues, L Santos Filho, MJG Mello, MD Asensi, MS Pereira, PP Gontijo Filho, M Rocha, RS Kuchenbecker, ES Medeiros, ACC Pignatari

**Affiliations:** 1 Escola de Enfermagem, USP - Universidade de São Paulo, São Paulo, Brazil; 2 Tropical Diseases, Faculdade de Medicina de Botucatu - UNESP - Univ Estadual Paulista, Botucatu, Brazil; 3 Laboratório Especial de Microbiologia Clínica, UNIFESP - Universidade Federal de São Paulo, São Paulo, Brazil; 4 UFRGS - Universidade Federal do Rio Grande do Sul, Porto Alegre, Brazil; 5 UFPA - Universidade Federal do Pará, Belém, Brazil; 6 UFC - Universidade Federal do Ceará, Fortaleza, Brazil; 7 UFPB - Universidade Federal da Paraíba, João Pessoa, Brazil; 8 IMIP - Pernambuco, Recife, Brazil; 9 FIOCRUZ - Rio de Janeiro, Rio de Janeiro, Brazil; 10 UFGO - Universidade Federal de Goiás, Goiânia, Brazil; 11 UFU - Universidade Federal de Uberlândia, Uberlândia, Brazil; 12 UNIFESP - Universidade Federal de São Paulo, São Paulo, Brazil

## Introduction

Minimal structure is required for an effective prevention of Healthcare-Associated Infection (HAI).

## Objectives

Countrywide evaluation of structure for HAI prevention in a sample of hospitals from Brazil.

## Methods

Hospitals from five Brazilian regions were evaluated (n=91; total of 8,853 beds). A team of trained nurses carried out the evaluation, focusing on structure and process issues for HAI prevention and control (HAI-PCC). Teaching hospitals were used as reference for comparisons.

## Results

The majority of components of HAI-PCC in reference hospitals were identified with conformity indexes (CI) above 80%; other hospitals have achieved CI below 70% in most situations. The component of HAI surveillance showed the worst CI in non-reference hospitals (below 65.7%). The worst ratio of beds/sinks was found in hospital with >200 beds (4.5, p<0.0001). Regarding alcoholic products, the worst ratio beds/dispensers was found in non reference hospitals with > 200 beds (4.2; p<0.001) or with <50 beds (4.0; p <0.001) compared to reference hospitals (2.8). The overall CI for handwashing structure was 51.7% and for hand hygiene with alcohol was 81.2%; better CI occurred in reference hospitals (65.2% and 83.1%, respectively). The CI for sterilization services showed huge variation from 0 to 100%, and was better for reference hospitals. Those hospitals were also more likely to have their own microbiology laboratories.

## Conclusion

These data point out to problems with structure for infection control in non-teaching hospitals in Brazil.

## Competing interests

None declared.

